# Bile salt hydrolase-overexpressing *Lactobacillus* strains can improve hepatic lipid accumulation *in vitro* in an NAFLD cell model

**DOI:** 10.29219/fnr.v64.3751

**Published:** 2020-11-12

**Authors:** Wenli Huang, Guangqiang Wang, Yongjun Xia, Zhiqiang Xiong, Lianzhong Ai

**Affiliations:** Shanghai Engineering Research Center of Food Microbiology, School of Medical Instrument and Food Engineering, University of Shanghai for Science and Technology, Shanghai, China

**Keywords:** Lactobacillus, bile salt hydrolase, non-alcoholic fatty liver disease, hepatic lipid accumulation, HepG2 cell, cholesterol

## Abstract

**Background:**

Non-alcoholic fatty liver disease (NAFLD) includes a range of liver diseases that occur in the absence of significant alcohol consumption. The probiotic bacterial strains *Lactobacillus casei* LC2W, which overexpresses the bile salt hydrolase (BSH) gene (referred to as pWQH01), and *Lactobacillus plantarum* AR113, which exhibits high BSH activity, have been shown to improve hepatic lipid accumulation and may lower cholesterol levels *in vivo*. These effects may be BSH-dependent, as *L. casei* LC2W without BSH activity did not exert these beneficial effects.

**Objective:**

This study aimed to investigate the effects of *Lactobacillus* with high BSH activity on cholesterol accumulation and lipid metabolism abnormalities in oleic acid (OA)- and cholesterol-induced HepG2 cell models, and to determine the mechanism underlying the effects.

**Design:**

A HepG2 cell model of OA-induced steatosis and cholesterol-induced cholesterol accumulation was developed. OA- and cholesterol-treated HepG2 cells were incubated with *L. plantarum* AR113, *L. casei* LC2W or *L. casei* pWQH01 for 6 h at 37°C with 5% CO_2_. Subsequently, a series of indicators and gene expressions were analysed.

**Results:**

Both *L. plantarum* AR113 and L. *casei* pWQH01 significantly reduced lipid accumulation, total cholesterol (TC) levels and 3-hydroxy-3-methyl-glutaryl-coenzyme A reductase (HMGCR) mRNA expression relative to the control group, whereas L. casei LC2W had no similar effect. Additionally, exposure to *L. plantarum* AR113 or *L. casei* pWQH01 significantly reduced the expression of sterol regulatory element-binding protein 1c (SREBP-1c), Acetyl-CoA carboxylase (ACC), fatty acid synthase (FAS) and tumour necrosis factor-α (TNF-α) andsignificantly increased the expression of 5' adenosine monophosphate-activated protein kinase (AMPK) and peroxisome proliferator-activated receptor alpha (PPARα).

**Conclusion:**

Both *L. plantarum* AR113 and *L. casei* pWQH01 appear to improve steatosis *in vitro* in a BSH-dependent manner.

## Popular scientific summary

*Lactobacillus plantarum* AR113 and *Lactobacillus casei* pWQH01 with high bile salt hydrolase active can improve steatosis by reducing the expression of SREBP-1c, ACC, FAS and TNF-α and increasing the expression of AMPK and PPARα *in vitro.*

Non-alcoholic fatty liver disease (NAFLD) is a clinicopathological syndrome characterised by hepatic steatosis at an alcohol consumption level of <20 g per day (1). The global incidence of NAFLD continues to increase and thus parallels the increasing prevalence of obesity, hyperlipidaemia, type 2 diabetes and metabolic syndrome (2–4). Several previous studies demonstrated that hepatic steatosis occurs due to increased lipogenesis and reduced lipid export from the liver. Therefore, the hepatic accumulation of neutral lipids derived from an imbalance in lipid acquisition and clearance is a hallmark of NAFLD. During NAFLD, hepatocytes exhibit altered cholesterol metabolism, which is characterised by the activation of cholesterol biosynthesis pathways, enhancement of cholesterol desertification and reduction in the cholesterol output and bile acid synthesis (5, 6). Currently, NAFLD treatment is limited to a restricted energy intake, increased physical activity and pharmacological treatments. However, these interventions often have unsatisfactory effects (7). The pathogenesis of NAFLD is often referred to as a ‘two hit’ pathophysiological theory. The ‘first hit’ is the deposition of free fatty acids (FFAs) in hepatocytes (i.e. steatosis), while the ‘second hit’ is the progression of steatosis to nonalcoholic steatohepatitis (NASH) (8). This progression is often associated with the release of cytokines, and a previous study identified a central role for the overexpression of pro-inflammatory cytokine tumour necrosis factor (TNF)-α mRNA in the aetiology of NASH (9). Human liver steatosis is associated with the accumulation of excess oleic acid (OA), a monosaturated omega-9 fatty acid and end product of *de novo* fatty acid synthesis (10). Morphologically, OA-treated human hepatoblastoma HepG2 cells and steatotic hepatocytes are similar (11).

Probiotics are the most widely used enteric microecological preparations in clinical settings. These microbes colonise the intestinal and reproductive systems of the body and can promote the microecological balance in the host, thereby improving disease symptoms and promoting health (12). Internationally, effective and complete probiotic strains must conform to the following four criteria: (1) beneficial to the host, (2) normal colonisation at a specific location in the host, (3) growth and survival at a specific location in/on the host and (4) maintenance of viability and activity during production and storage (13). Commonly used probiotics can be classified as *Lactobacillus*, *Bifidobacterium*, Gram-positive cocci and some fungi (14). The 2015 Recommended Guide for Probiotics Application identified the following probiotic preparations as preferred for the treatment of NAFLD: *Lactobacillus plantarum*, *Lactobacillus rhamnosus*, *Bifidobacterium bifidum*, *Streptococcus thermophilus* and *Bifidobacterium longum*. Many studies have shown that probiotics can improve liver function, blood lipid profiles, insulin resistance, inflammatory factors and even the degree of liver fibrosis in NAFLD patients, indicating that these agents may have potential therapeutic value in this population (15, 16). Recent studies have demonstrated that bile acids can significantly impact many disease states, including NAFLD (16, 17). The gut microbiota may affect bile acid metabolism by modulating a critical step catalysed by bile salt hydrolases (BSHs), which hydrolyse the amide bond and release glycine or taurine from the steroid cores of primary bile acids. The forming deconjugated bile acids can undergo different subsequent transformations that may affect the physiology and disease status of the host (17), so many researchers speculate that BSH may play an important role. BSH is an important gatekeeper to subsequent bile acid transformations, but it remains unclear whether high BSH activity can relieve NAFLD and if so, by which mechanism.

In our previous work, we observed that *L. plantarum* AR113 (high BSH activity) and *L. casei* pWQH01 which overexpresses BSH from *L. plantarum* AR113 (high BSH activity) ameliorated cholesterol accumulation *in vivo,* whereas *L. casei* LC2W (no BSH activity) had no obvious effect on hypercholesterolemia (18). This study aimed to investigate the beneficial effects of *Lactobacillus* strains with high BSH activity on cholesterol accumulation and lipid metabolism abnormalities *in vitro*, using OA- and cholesterol-induced HepG2 cells as a model for NAFLD, and to subsequently determine the underlying mechanism.

## Materials and methods

### Bacterial strains, growth conditions and treatments

*L. plantarum* AR113, *L. casei* pWQH01 and *L. casei* LC2W strains were obtained from the Shanghai Engineering Research Centre of Food Microbiology, University of Shanghai for Science and Technology (Shanghai, China). *L. casei* LC2W does not harbour the *bsh1* gene and thus lacks BSH activity. *L. casei* pWQH01 overexpresses *bsh1*, and this gene is also responsible for the high level of BSH activity in *L. plantarum* AR113. The AR113, pWQH01 and LC2W strains were stored at -80°C in de Man, Rogosa and Sharpe (MRS) broth containing 20% glycerol. The strains were activated three successive times in sterile MRS broth (Difco, Detroit, MI, USA) and incubated at 37°C for 24 h. Prior to experimental use, a 2% (v/v) inoculum of each strain was sub-cultured and incubated at 37°C for 24 h. Each probiotic suspension was adjusted to a concentration of 1 × 10^9^ CFU/mL. Next, the probiotic suspensions were centrifuged at 4,000 rpm for 5 min, and the pellets were washed with phosphate buffered saline (PBS) and resuspended in non-antibiotic supplemented cell culture medium.

### Oleic acid/BSA complex solution preparation

An oleic acid/Bovine serum albumin (OA/BSA) complex solution was prepared as previously described, with minor modifications (19). First, a 100 mM OA stock solution was prepared in 0.1 N NaOH by heating to 70°C in a shaking water bath. Next, a 10% (w/v) BSA solution was prepared in H_2_O by heating to 55°C in a shaking water bath. Finally, a 10 mM OA solution containing 10% BSA was diluted in culture medium to obtain the desired final concentrations. The OA/BSA complex solution was sterile-filtered through a 0.45-μm membrane filter and stored at -20°C.

### Cell culture and treatment

The human hepatocellular liver carcinoma cell line, HepG2, was purchased from the Cell Resources Centre of Shanghai Institutes for Biological Sciences (Shanghai, China). Briefly, cells were cultured in Dulbecco's Modified Eagle Medium (DMEM) containing 10% fetal bovine serum (FBS), 100 U/mL penicillin and 100 U/mL streptomycin in an incubator at 37°C under a humidified atmosphere of air containing 5% CO_2_ until an ~80% confluent monolayer was obtained. All cell culture components were purchased from Gibco™ (Thermo Fisher Scientific, Grand Island, NY, USA). HepG2 monolayers were pre-incubated with non-antibiotic supplemented cell culture medium for 6 h before further assays. A HepG2 cell model of OA-induced steatosis and cholesterol-induced cholesterol accumulation was developed (20). Cells were seeded at a density of 5 × 10^5^ cells/mL in six-well plates and cultured in FBS-free medium for 24 h in triplicate, after which the cells were induced by exposure to 1 mM OA and 75 μg/mL water-soluble cholesterol (Sigma-Aldrich, Saint Louis, MO, USA) for 6 h. Control group cells were treated with OA‑free and cholesterol-free medium containing 1% BSA. Finally, 1 mL of each probiotic suspension was added to the OA- and cholesterol-treated HepG2 cells, which were incubated for 6 h at 37°C with 5% CO_2_. A series of experimental analyses were performed as described below.

### Cell viability assay

To assess cell viability, 5 × 10^4^ HepG2 cells in 100 μL were seeded in 96-well plates and cultured overnight. Next, the HepG2 cells were treated with *L. plantarum* AR113, *L. casei*LC2W or *L. casei* pWQH01 and a mixture of 1 mM OA with 50 μg/mL cholesterol, 1 mM OA with 75 μg/mL cholesterol or 1 mM OA with 100 μg/mL cholesterol for 2, 4, 6 or 8 h under the same culture conditions described above. Finally, cell viability was measured using a Cell Counting Kit-8 (CCK-8) assay kit (Beyotime, Shanghai, China) as per the manufacturer’s instructions (*n* = 3).

### Oil Red O staining of HepG2 cells

After treatment with each of the probiotic suspensions for 6 h, HepG2 cells were carefully washed three times with cold PBS to avoid disruption and subsequently fixed with 10% formalin solution for 20 min at room temperature. The fixed cells were then stained with a freshly prepared working solution of Oil Red O (Nanjing Jianchen Bioengineering Institute, Jiangsu, China) in the dark for 15 min. Next, the stained cells were washed several times with cold distilled water and observed and imaged under an inverted microscope (Nikon, Tokyo, Japan). The intracellular lipid-bound stain was re-dissolved in 100% isopropanol and shaken at room temperature for 10 min, after which the resultant solution was transferred to a 96-well plate. The optical density of the resultant solution was read at a wavelength of 510 nm.

### Intracellular cholesterol content of HepG2 cells

After exposing the cells to *L. plantarum* AR113, *L. casei* LC2W or *L. casei* pWQH01 for 6 h, the culture media was removed carefully without disturbing the cells. The cells were gently washed with cold PBS, lysed in radioimmunoprecipitation assay (RIPA) buffer and homogenised. The homogenate was centrifuged at 13,000 rpm and 4°C for 20 min to remove the insoluble materials, after which the intracellular cholesterol content was normalised against the total cellular protein content using a BCA protein assay kit (Beyotime, Shanghai, China).

### Cholesterol uptake assay

Cholesterol uptake was analysed by comparing HepG2 cells treated with the viable probiotic strains, the mixture of OA and cholesterol or no treatment. The cholesterol concentrations in the cells were measured using the modified *0*-pthalaldehyde colorimetric method, as described by Gilliland (21). Briefly, 0.25 mL of homogenate was mixed with 1.5 mL of 95% (v/v) ethanol and 1 mL (50%, w/v) of KOH, vortexed for 15 s and heated at 60°C in a water bath for 10 min. After cooling the mixture to room temperature, 2.5 mL of hexane and 1.5 mL of distilled water were added and the resultant mixture was vortexed. The hexane layer was transferred into a new glass tube and evaporated under a nitrogen gas flow at 60°C. Next, 0.75 mL of *0*-phthalaldehyde reagent (0.05 g *0*-pthalaldehyde dissolved in 100 mL glacial acetic acid) was immediately added to the residue. Finally, 1 mL of concentrated H_2_SO_4_ was added, and the resultant mixture was mixed thoroughly and incubated at room temperature for a further 10 min before reading the absorbance at 550 nm. A standard curve was generated using solutions of 0, 0.02, 0.05, 0.10, 0.12, 0.15 and 0.20 mg/mL cholesterol (*R*^2^= 0.9992). All the above-listed organic reagents were purchased from the Beijing Land Bridge Technology Co., Ltd. (Beijing, China).

### Analysis of lipid-regulating genes in HepG2 cells by qRT-PCR

Following treatment with each of the three probiotic strains for 6 h, the cells were gently washed twice with cold PBS and lysed in RIPA buffer, after which the total RNA was extracted as per the manufacturer’s instructions. The amount of RNA was determined, and the purity was checked by measuring the optical densities at 260 and 280 nm. Next, cDNA was synthesised from the isolated total RNA using a cDNA RT PreMix kit (TaKaRa Bio, Otsu, Japan) as per the manufacturer’s protocol, and subjected to quantitative reverse transcription polymerase chain reaction (qRT-PCR) using SYBR Premix Ex TaqII (TaKaRa Bio, Otsu, Japan) on a LightCycler^®^ 96 (Roche, Basel, Switzerland). PCR amplification was performed using following conditions: 95°C for 30 s, followed by 40 cycles of 95°C for 5 s and 60°C for 30 s. The primer sequences (Sangon Biological Engineering, Shanghai, China) used in this study are shown in Table 1. Gene expression was normalised to the glyceraldehyde 3-phosphate dehydrogenase (GAPDH) mRNA levels. The transcript levels in each group were determined using the 2^−(∆∆C^^t^^)^ method (22) and are expressed as a ratio of expression relative to the control group.

**Table 1:** Specific human gene primer sequences

Gene name	Primer sequences
GAPDH	Forward 5**'**-CGCTCTCTGCTCCTCCTGTT-3**'**
	Reverse 5**'**-CCATGGTGTCTGAGCGATGT-3**'**
AMPK	Forward 5**'**-AGGATGCCTGAAAAGCTTGA -3**'**
	Reverse 5**'**-GACAGCCGGAGAAGCAGAAAC-3**'**
SREBP-1c	Forward 5**'**-GCGGAGCCATGGATTGCAC-3**'**
	Reverse 5**'**-TCTTCCTTGATACCAGGCCC-3**'**
ACC	Forward 5**'**-TGTCTGAAGAGATTAGGGAAGT-3**'**
	Reverse 5**'**-GTTATGTGAAAGATGTGGATGA-3**'**
FAS	Forward 5**'**-TTCGTTTGTGAGCCTGACTGC-3**'**
	Reverse 5**'**-GCTCCCGGATCACCTTCTTG-3**'**
PPARα	Forward 5**'**-TCCGACTCCGTCTTCTTGAT-3**'**
	Reverse 5**'**-GCCTAAGGAAACCGTTCTGTG-3**'**
HMGCR	Forward 5**'**-GACCTTTCCAGAGCAAGCAC-3**'**
	Reverse 5**'**-TCAACAAGAGCATCGAGGGT-3**'**
TNFα	Forward 5**'**-CAGCCTCTTCTCCTTCCTGAT-3**'**
	Reverse 5**'**-GCCAGAGGGCTGATTAGAGA-3**'**

### Statistical analyses

All results are expressed as means ± standard deviations. Between-group differences were assessed using a one-way analysis of variance (ANOVA) with Tukey’s multiple comparison test. Statistical analyses were performed using the Statistical Package for the Social Sciences (SPSS) statistical software package, version 20 (IBM, Armonk, NY, USA), and a *p* value < 0.05 was considered statistically significant.

## Results

### Cytotoxic effects of probiotic strains and combined OA and cholesterol treatment on HepG2 cells

Treatment of HepG2 cells with the various probiotic strains (*L. plantarum* AR113, *L. casei* LC2W and *L. casei* pWQH01) for 2, 4, 6 or 8 h did not cause any cytotoxicity. However, the cell viability was greatly reduced after an 8-h co-incubation, compared to a 6-h co-incubation. Therefore, 6 h was selected as the endpoint for subsequent experiments.

**Fig. 1:**
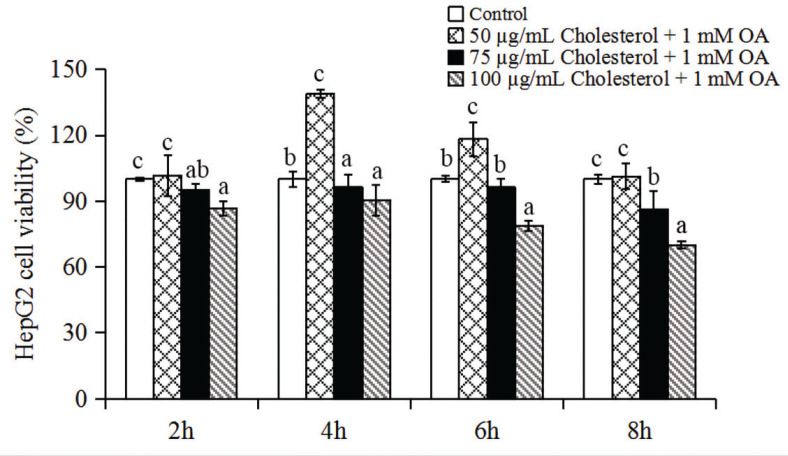
The effects of different concentrations of cholesterol and 1 mM oleic acid (OA) on the viability of HepG2 cells after treatment for 2, 4, 6 and 8 h. Data represent the means ± standard deviations (*n* = 3). Statistical differences between groups were determined by a one-way ANOVA. Different letters indicate statistically significant differences between groups, *P* < 0.05.

HepG2 cells were treated with a mixture of 1 mM OA plus 50, 75 or 100 μg/mL cholesterol for 2, 4, 6 or 8 h to induce hepatic steatosis. We estimated the impact of each treatment on cell survival based on the following cell viability criteria (23, 24): >90%, unaffected; 80–90%, moderately affected and <80%, cytotoxicity of the compound(s). The mixture of OA and cholesterol suppressed the proliferation of HepG2 cells in a dose-dependent manner (Fig. 1). Cells treated with 1 mM OA plus 50, 75 or 100 μg/mL cholesterol for 6 h yielded viability values of 118%, 93% and 80%, respectively. The viability of cells treated with 75 μg/mL cholesterol and 1 mM OA did not differ significantly when compared to the control group. Therefore, 75 μg/mL cholesterol and 1 mM OA were used to induce hepatic steatosis in subsequent experiments.

### Probiotic strains with high BSH activity reduced lipid accumulation and lipid content of HepG2 cells

**Fig. 2:**
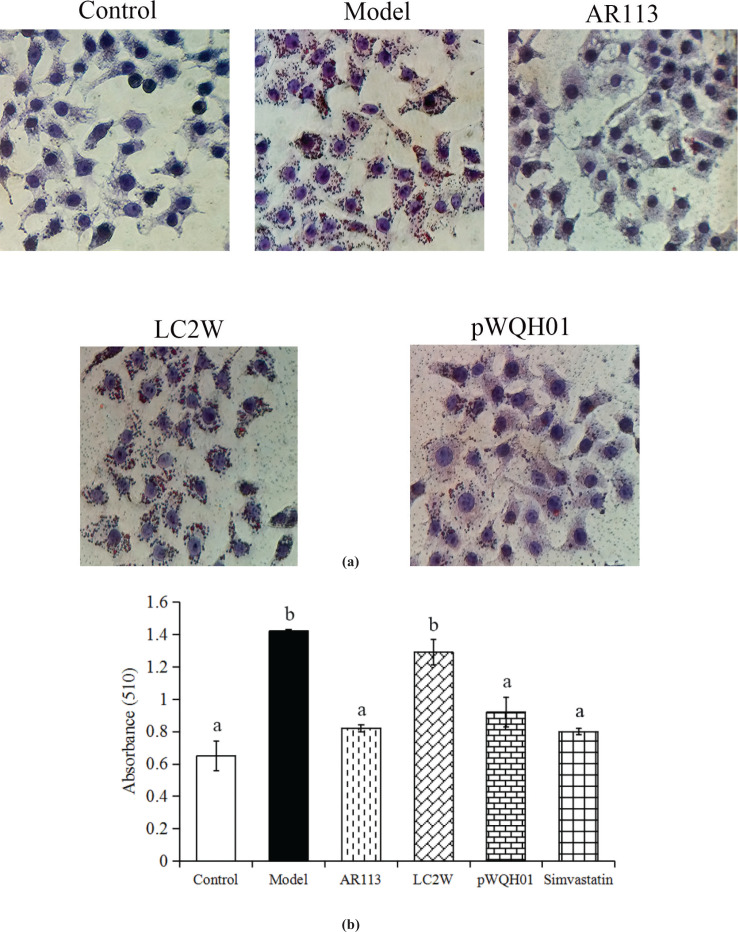
The effects of probiotic strains on lipid accumulation and lipid contents in HepG2 cells. (a) After treatment with probiotic strains for 6 h, lipids were stained with Oil Red O (400× magnification). (b) Spectrophotometric quantification of the total lipid contents by dissolving the stained oil droplets from HepG2 cells. Data represent the means ± standard deviations (*n* = 3). Statistical differences between groups were determined by a one-way ANOVA. Different letters indicate statistically significant differences between groups, *P* < 0.05.

Oil Red O staining was used to determine the effects of the probiotic strains on cellular lipid accumulation. *L. casei* LC2W and *L. casei* pWQH01 differ only in terms of BSH activity; the former has no BSH activity, while the latter overexpresses *L. plantarum* AR113 *bsh1* and exhibits high BSH activity. Oil Red O staining indicated that HepG2 cells in the control group did not experience significant steatosis, whereas cells in the OA and cholesterol-treated group exhibited severe steatosis. Cells treated with *L. plantarum* AR113 and *L. casei* pWQH01 exhibited significantly decreased lipid accumulation relative to the OA- and cholesterol-treated cells. In contrast,*L. casei* LC2W treatment did not significantly reduce the intracellular lipid content (Fig. 2a). *L. plantarum* AR113 and *L. casei* pWQH01 also significantly decreased the total lipid content relative to the OA- and cholesterol-treated groups, as reflected by a spectrophotometric analysis; again, *L. casei* LC2W had no significant effect (Fig. 2b).

### Probiotic strains with high BSH activity reduced the TC levels in HepG2 cells by attenuating HMGCR expression

**Fig. 3:**
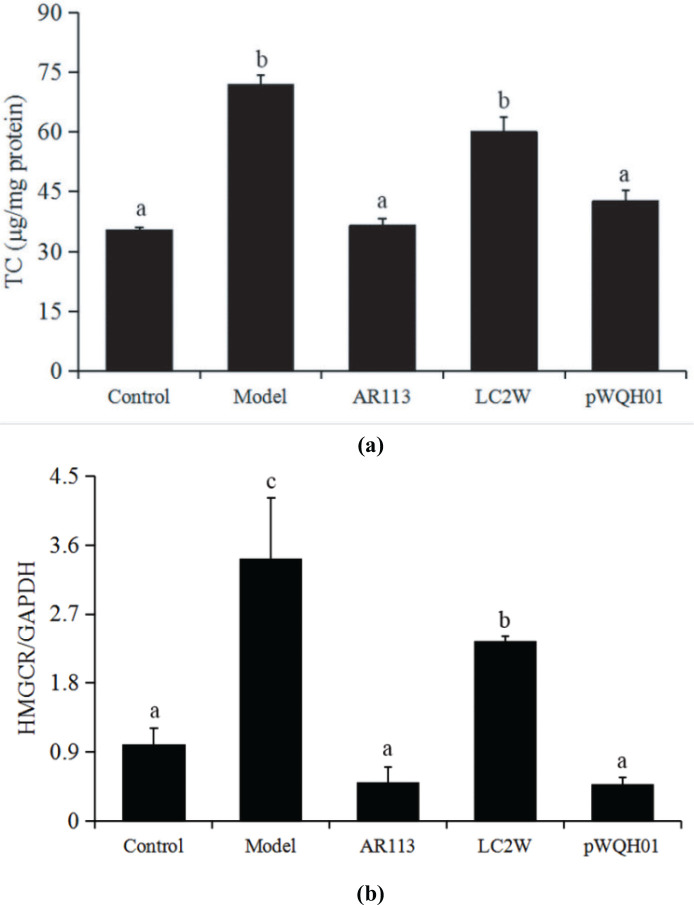
The effects of probiotic strains on total cholesterol (TC) levels. (a) TC levels in HepG2 cells. (b) HMGCR mRNA levels. Data represent the means ± standard deviations (*n* = 3). Statistical differences between groups were determined by a one-way ANOVA. Different letters indicate statistically significant differences between groups, *P* < 0.05.

**Fig. 4:**
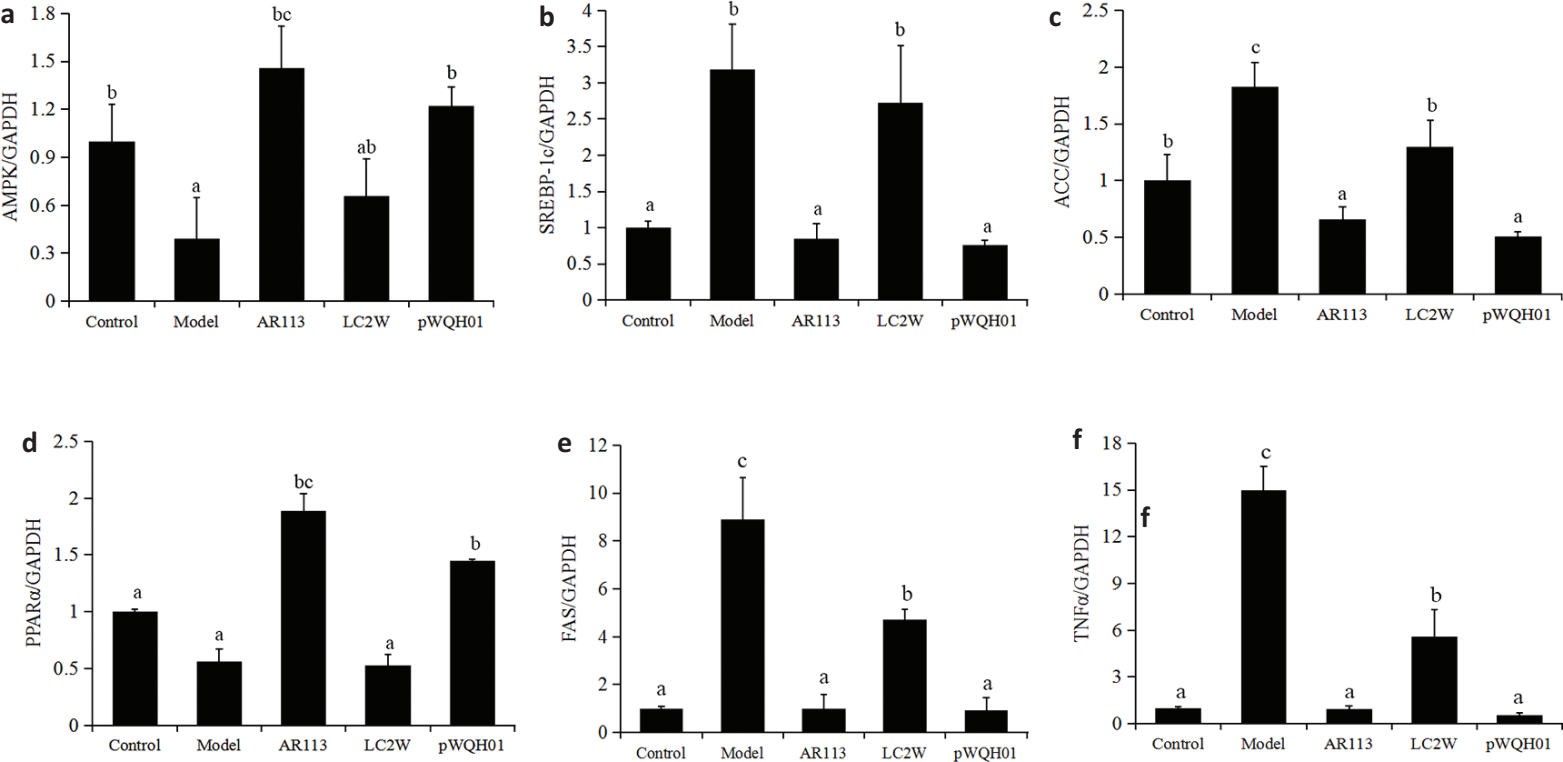
The effects of probiotic strains on the mRNA expression of key lipid-regulating genes in HepG2 cells stimulated with oleic acid OA and cholesterol. (a) AMPK, (b) SREBP-1c, (c) ACC, (d) PPARα, (e) FAS and (f) TNFα. Data represent the means ± standard deviations (*n* = 3). Statistical differences between groups were determined by a one-way ANOVA. Different letters indicate statistically significant differences between groups, *P* < 0.05.

Recent studies showed that disordered cholesterol metabolism can lead to the processes of cellular lipid accumulation and hepatic dysfunction, which are closely related to the development of NAFLD (25, 26). Figure 4a demonstrates significant increases of more than twofold in the TC levels of OA- and cholesterol-treated cells relative to the untreated controls (*P* < 0.05). Promisingly, the cells treated with *L. plantarum* AR113 or *L. casei* pWQH01 resembled control cells, with significantly (*P* < 0.05) lower levels of TC accumulation than cells in the OA- and cholesterol-treated group, whereas *L. casei* LC2W-treated cells had levels similar to those of OA- and cholesterol-treated cells (Fig. 3a). These results are consistent with the above-described results of Oil Red O staining.

HMGCR is a rate-limiting enzyme in the metabolic pathway that produces cholesterol and other isoprenoids, and is a frequent target of cholesterol-lowering drugs. Figure 3b shows that HMGCR expression increased significantly (*P* < 0.05) in the OA and cholesterol-treated group by almost fourfold. In contrast, cells treated with *L. plantarum* AR113 or *L. casei* pWQH01 exhibited significantly (*P* < 0.05) decreased HMGCR expression, with levels below those detected in controls. Notably, HMGCR expression was much higher in *L. casei* LC2W-treated cells than in cells treated with *L. plantarum* AR113 or *L. casei* pWQH01 or even the control group. These findings indicate that some probiotic strains may suppress HMGCR expression by altering BSH activity.

### Probiotic strains modulate key lipid-regulating genes in HepG2 cells

To investigate the mechanism by which probiotic strains improve hepatic lipid accumulation in HepG2 cells, we monitored the mRNA expression of vital lipid-regulating genes in various treatment groups. Notably, we observed significantly higher expression of SREBP-1c, ACC, FAS and TNFα mRNA and significantly lower expression of AMPK and PPARα mRNA in OA- and cholesterol-treated cells relative to control group cells (Fig. 4). However, exposure to *L. plantarum* AR113 or *L. casei* pWQH01 significantly reduced the expression of SREBP-1c, ACC, FAS and TNFα mRNA and significantly increased the expression of AMPK and PPARα mRNA in OA- and cholesterol-treated cells. In contrast, no significant differences in the expression of AMPK, SREBP-1c, ACC and PPARα mRNA were observed between *L. casei* LC2W and OA- and cholesterol-treated cells (Fig. 4). These indicate that BSH activity level plays an important role in the probiotic-mediated modulation of key lipid-regulating genes in HepG2 cells.

## Discussion

NAFLD is a clinical pathological change characterised by the accumulation of triglycerides in hepatocytes and is among the most common causes of abnormal liver function tests (27). Recently, the incidence of NAFLD has increased significantly along with increases in the prevalence of dyslipidaemia, obesity and type 2 diabetes mellitus. According to one report, more than 10% of NAFLD patients progress to NASH, and some patients in the latter group eventually develop cirrhosis and hepatocellular carcinoma (28). Although some drugs have been developed to improve NAFLD, these induce many side effects (29). Therefore, new treatments for NAFLD/NASH are urgently needed.

In recent years, many studies have proposed the beneficial effects of probiotics on health (30, 31). Several lines of evidence have indicated the anti-obesity and hepatoprotective activities of probiotics (32). For example, probiotics can improve hepatic lipid metabolism and reduce serum triacylglycerol and total cholesterol (TC) levels in patients with NAFLD. For example, yogurt supplemented with *L. acidophilus* and *Bifidobacterium*spp. can reduce the serum levels of ALT, AST, TC and low-density lipoprotein cholesterol in patients with NAFLD (33). However, the roles of these probiotic strains and the molecular mechanism(s) underlying the alleviation of NAFLD pathogenesis remain poorly understood. In this study, we investigated the beneficial effects of *L. plantarum* AR113 and *L. casei* pWQH01, which exhibit high BSH activity, on cholesterol levels and hepatic lipid accumulation *in vitro*, as well as the mechanism underlying these effects.

Previous studies have demonstrated the considerable involvement of cholesterol metabolism in the development of hepatic lipid accumulation and cellular dysfunction, which are closely related to the development of NAFLD (25, 26). Our results demonstrated that the treatment of OA- and cholesterol-treated HepG2 cells with *L. plantarum* AR113 and *L. casei* pWQH01 led to significant decreases in the TC levels and HMGCR mRNA expression when compared with the OA and cholesterol treatment alone. In contrast, treatment with *L. casei* LC2W had no significant effects relative to OA and cholesterol treatment alone. As *L. plantarum* AR113 and *L. casei* pWQH01 exhibit high BSH activity while *L. casei* LC2W has no BSH activity, our results suggest that BSH activity may be responsible for the beneficial effects of the former strains against NAFLD. These findings are consistent with those of previous studies (34, 35), which demonstrated that probiotics with BSH activity can reduce cholesterol levels and serum lipid parameters (TC and low-density lipoprotein cholesterol levels) (34, 35). Our Oil Red O staining examination revealed a marked intracellular accumulation of lipids in the HepG2 cell model of NAFLD (OA- and cholesterol-treated cells). The total lipid and TC levels were lower in the *L. plantarum* AR113 and *L. casei* pWQH01 groups than in the OA- and cholesterol-treated and *L. casei* LC2W-treated groups. These results show that exposure to *L. plantarum* AR113 and *L. casei* pWQH01 can significantly prevent hepatic lipid accumulation by reducing cholesterol synthesis in a BSH-dependent manner.

To further investigate the mechanism by which *L. plantarum* AR113 and *L. casei* pWQH01 ameliorate hepatic lipid accumulation, we quantitatively analysed the expression of key genes involved in fatty acid synthesis and metabolism using qRT‑PCR. Steatosis is caused primarily by an increased flux of fatty acids to the liver, which is consequent to the high availability of plasma FFAs relative to the peripheral oxidative requirements (20). AMPK is an energy sensor that regulates hepatic lipogenesis and may thus be a therapeutic target in the treatment of fatty liver disease (36). Once activated, AMPK inhibits ACC expression by down-regulating SREBP-1c and thus attenuates hepatic steatosis (37). SREBP‑1c and FAS are also involved in fatty acid synthesis and the development and pathogenesis of NAFLD (38). In our study, OA- and cholesterol-treated cells exhibited significant increases in the expression of SREBP-1c, ACC and FAS mRNA that paralleled a decrease in the expression of AMPK mRNA. This finding indicates a negative feedback loop that regulates SREBP-1c expression via AMPK upon exposure to OA and cholesterol. Compared with the untreated model cells, cells treated with *L. plantarum* AR113 and *L. casei* pWQH01 exhibited significant decreases in the expression of SREBP-1c, ACC and FAS mRNA and a significantly increase in AMPK mRNA, whereas no significant changes were observed upon exposure to *L. casei* LC2W. In other words, *L. plantarum* AR113 and *L. casei* pWQH01 appear to reduce hepatic lipid accumulation by targeting AMPK-mediated fatty acid synthesis and thus inhibiting SREBP‑1c, ACC and FAS expression in a BSH-dependent manner.

PPARα induces the expression of various target genes involved in the uptake, transportation and β-oxidation of fatty acids (39). PPARα also augments fatty acid catabolism and thus may protect against liver fat deposition (40). In this study, OA- and cholesterol-induced steatosis led to a decrease in PPARα expression in HepG2 cells, suggesting that this receptor may play a pathogenic role in NAFLD. Our results demonstrated an increase in the expression of PPARα after treatment with *L. plantarum* AR113 or *L. casei* pWQH01. The results may also indicate that *L. plantarum* AR113 and *L. casei* pWQH01 can exploit BSH to reduce liver fat deposition by decreasing lipid peroxidation, which may be mediated by enhanced PPARα expression.

Several studies have observed the overexpression of TNFα mRNA in both the liver and adipose tissues of severely obese patients with NASH (41). An elevated TNFα level plays a key role in the pathogenesis and disease progression of NAFLD (42). Previous studies have reported that the treatment of HepG2 cells with FFA may upregulate the expression of TNFα mRNA (43). In our study, we confirmed that OA treatment significantly upregulated the expression of TNFα mRNA in HepG2 cells, whereas exposure to *L. plantarum* AR113 and *L. casei* pWQH01 significantly decreased this expression. Our data further support a pathogenic role for hepatocyte-derived TNFα in NAFLD. However, it remains to be determined whether the increased TNFα in NAFLD is derived from hepatocytes or other inflammatory cells.

## Conclusions

Taken together, our data suggest *L. plantarum* AR113 and *L. casei* pWQH01, which both exhibit high BSH activity, might protect the liver from NAFLD and decrease hepatic lipid accumulation by reducing fatty acid synthesis. These beneficial effects may be mediated via the modulation of cholesterol synthesis and lipid-regulating gene expression in a BSH-dependent manner. *L. casei* LC2W, which lacks BSH activity, cannot mediate these effects. Our results indicate that BSH plays an important role in the beneficial effects of *Lactobacillus* strains in the treatment of NAFLD. In the future, probiotics with high BSH activity should be considered as potentially beneficial for the treatment of NAFLD.
